# Combined Chiari Malformation Type I and Syringohydromyelia in a Patient With Intractable Headache

**DOI:** 10.7759/cureus.63127

**Published:** 2024-06-25

**Authors:** Dhaval Trivedi, Byasha Jahangir, Sara Hasan, Franklyn Fenton

**Affiliations:** 1 Internal Medicine, New York Presbyterian-Brooklyn Methodist Hospital, Brooklyn, USA

**Keywords:** neurology, chiari malformation, syringomyelia, spinal cord, syringohydromyelia

## Abstract

Chiari malformations (CM) are a spectrum of hindbrain abnormalities involving the cerebellum, brainstem, skull base, and cervical cord. The most common is Chiari I malformation, in which the cerebellar tonsils descend through the foramen magnum. As opposed to types II-IV, which are congenital, type I can manifest in late childhood or adulthood with headaches and focal neurological symptoms. It can be caused by genetic variation, conditions that alter the basal skull or increase intracranial pressure, and even injury. Syringohydromyelia (SHM) is a neurological disorder characterized by longitudinal dilation of the central canal of the spinal cord with accumulated cerebrospinal fluid. This case report demonstrates a 35-year-old male with headaches, neck pain, back pain, and paresthesias who was found to have CM type-I malformation and syringohydromyelia.

## Introduction

Chiari malformation refers to a group of anatomical variations at the craniovertebral junction. Chiari malformation type 1 (CM1), the most common subtype, with a prevalence of 1%, describes the protrusion of the cerebellar tonsils through the foramen magnum [1]. Chiari malformation type 1 may be asymptomatic and diagnosed incidentally, but it can also present with Valsalva-induced headaches and other nonspecific symptoms. The pathophysiology suggests a morphology mismatch between a normally developed hindbrain and a small posterior cranial fossa, which inherently causes ectopia of the cerebellar tonsils [1]. The gold standard for diagnosis remains magnetic resonance imaging. Management of Chiari malformation type 1 is controversial, especially in adults, due to the rarity of the disease and a lack of clear guidelines. Surgical intervention is typically reserved for those with intractable headaches or neurological deficits from an associated syringomyelia [2].

Syringohydromyelia is a neurological disorder characterized by the longitudinal dilation of the central canal of the spinal cord with accumulated cerebrospinal fluid [3]. With a wide range of association (23-80%) with Chiari malformation type 1, there is increased discrepancy and possible overlap among cases of the true syrinx, central canal dilation, and cord edema or clinical presyrinx [4]. Further studies have demonstrated that despite the wide range of associative relationships between Syringohydromyelia and Chiari malformation type 1, the role of developmental foramen magnum compromise remains a statistically significant association [5].

Although several theories explain syringohydromyelia in Chiari malformation, the dominant theory suggests that occlusion of the subarachnoid spaces in the foramen magnum leads to the formation of the syrinx cavity by the movement of cerebrospinal fluid through perivascular and extracellular spaces in the spinal cord. There is ongoing debate regarding the management of Syringohydromyelia and Chiari malformation type 1 [6]. Besides pharmacological management of increased intracranial pressure, surgical interventions include posterior fossa decompression, intra-arachnoidal dissection, exploration of the fourth ventricle, or reduction of the cerebral tonsils. It is worth noting that surgical success depends on the ability to permanently establish a patent subarachnoid space at the foramen magnum [7].

## Case presentation

A 35-year-old male with a medical history of morbid obesity presents with intractable headaches, blurry vision, neck pain, and paresthesias. The onset of these symptoms was five days, two days, and one day before presentation, respectively. One week before admission, he visited the emergency department, where a CT brain scan revealed low-lying cerebellar tonsils, raising concerns for Chiari malformation. Given the improvement of his symptoms with pain medications at the time, the patient was discharged home with instructions to follow up with a neurologist in the outpatient setting. Two days later, he presented to a different emergency department with an intractable headache and new-onset right-sided facial and bilateral upper extremity numbness, accompanied by one episode of vomiting and photophobia. He was evaluated by neurology and was recommended for pain control, IV fluids, and outpatient neurology follow-up. The patient was once again discharged home.

**Figure 1:**
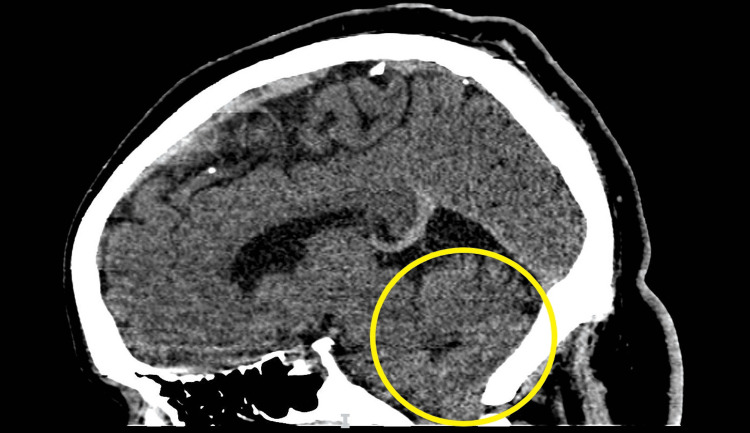
CT head demonstrating cerebellar tonsillar herniation (sagittal view) Yellow circle: Downward cerebellar tonsillar herniation extending below the foramen magnum

**Figure 2:**
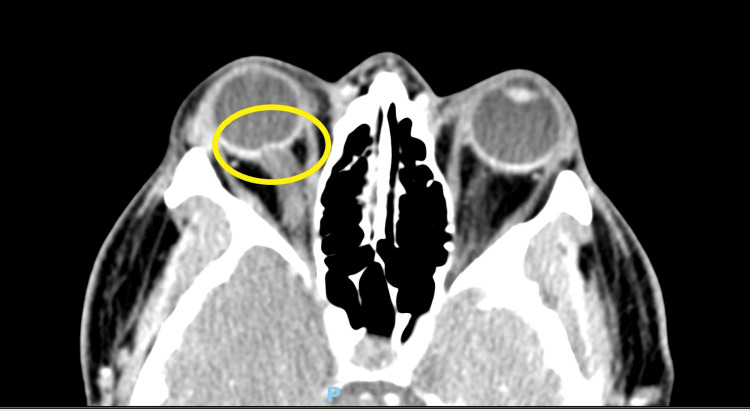
CT-head evaluating optic nerve tortuosity and posterior optic globe Yellow circle: CT-head demonstrating tortuosity of the optic nerves and flattening of the right posterior globe with probably invagination of the optic nerves through the optic cup

**Figure 3:**
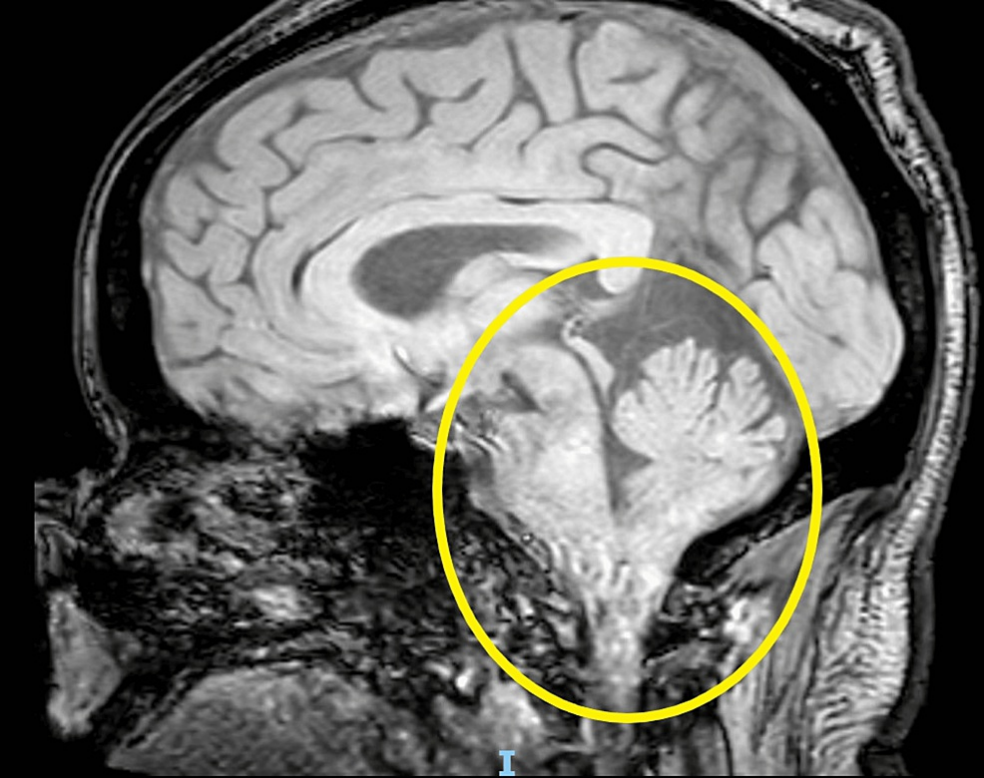
MRI brain (sagittal view) Yellow circle: gross cerebellar ectopia extending 1.5 cm below the foramen magnum on the right

**Figure 4:**
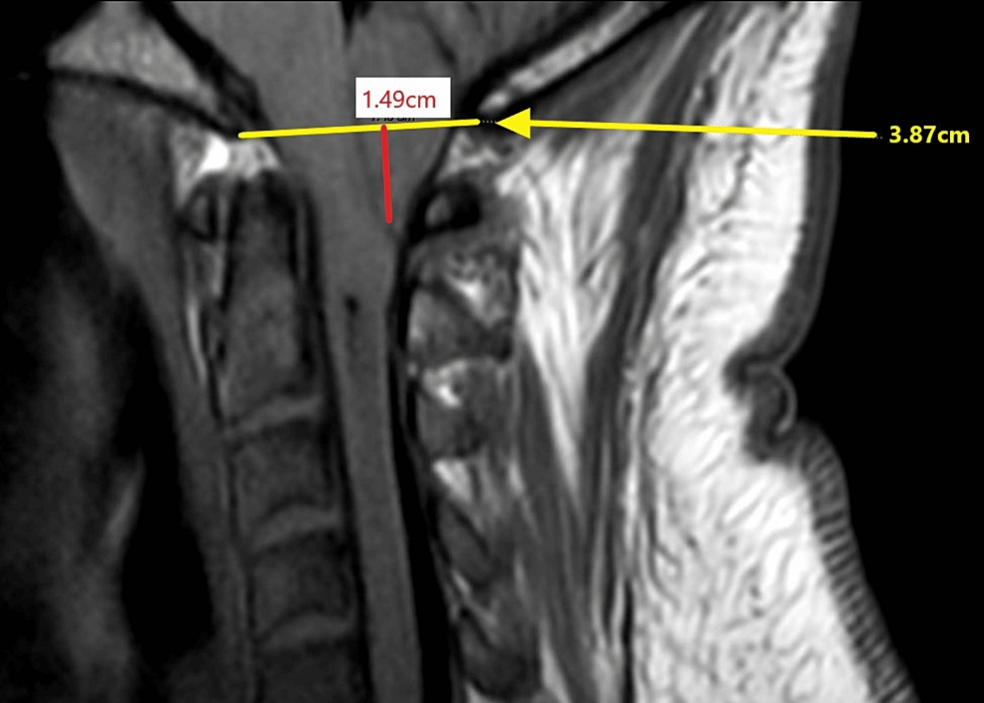
MRI cervical spine (sagittal view) demonstrating cerebellar ectopia Red line: Cerebellar ectopia extending 1.5 cm below the foramen magnum. Yellow line: Representing the level of the foramen magnum (3.87 cm in width)

Three days later, he returned to the emergency department due to ongoing symptoms, including a worsening intractable headache, and was noted to be in severe distress. He complained of an occipital headache described as a pressure-like sensation, rated 10/10 in intensity, along with persistent neck pain, blurry vision, and right-sided facial and bilateral upper extremity paresthesias. Vital signs at the time were significant for mild tachycardia and hypertension. A fundoscopic exam revealed bilateral papilledema. A neurological exam was notable for a partial right-sided facial sensory deficit in the V1-V3 distribution and a bilateral upper extremity sensory deficit. Visual acuity was intact on the neurological exam, although the patient complained of subjective bilateral blurry vision and partial vision loss in the right lower quadrant. A CT of the head demonstrated downward cerebellar tonsillar herniation extending up to 2.4 cm below the foramen magnum (Figure 1; yellow circle) and tortuosity of the optic nerves bilaterally, with flattening of the right posterior globe, probable invagination of the optic nerves through the optic cup, and dilation of the CSF sheath around the optic nerves (Figure 2; yellow circle). MRI of the head and cervical spine demonstrated gross bilateral cerebellar ectopia extending 1.5 cm below the foramen magnum on the right (Figures 3, 4; yellow circle).

**Figure 5:**
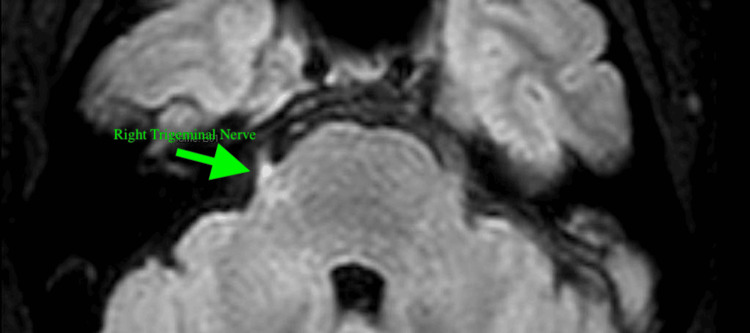
MRI brain evaluating right trigeminal nerve Green arrow: Symmetric T2 hyperintense signal at the root entry zone and cisternal segment of the right trigeminal nerve

**Figure 6:**
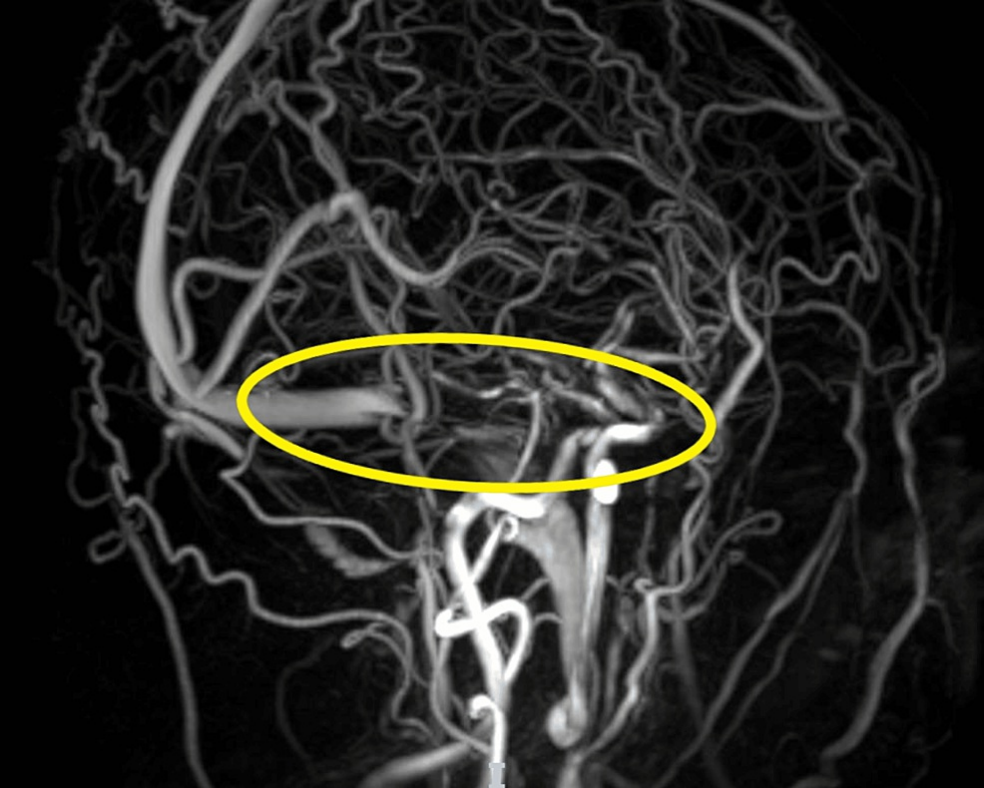
MR venogram with high grade right transverse sinus stenosis Yellow circle: High-grade stenosis of the dominant right transverse sigmoid sinus junction

He was evaluated by neurology and recommended for a lumbar puncture, which was unsuccessful, and the patient was hospitalized in the general medical ward for pain control and further evaluation. Several repeat attempts at lumbar puncture were unsuccessful, and interventional radiology deferred reattempts given the presence of Chiari I malformation. The patient was started on acetazolamide for presumed increased intracranial pressure, as well as pain medications. Neurosurgery was consulted, and further imaging was performed. An MRI of the brain and cervical spine revealed an asymmetric T2 hyperintense signal at the root entry zone and cisternal segment of the right trigeminal nerve (Figure 5; green arrow), and MRI venography of the head revealed high-grade stenosis at the right transverse sigmoid sinus junction and the non-dominant left transverse sigmoid sinus junction (Figure 6; yellow circle).

The patient’s headache and blurry vision partially improved following the administration of acetazolamide. However, given the imaging findings demonstrating high-grade venous sinus stenosis, he was initiated on dual antiplatelet therapy with aspirin and clopidogrel, with plans for venous sinus stenting the following day.

The patient was brought to the angiography suite and placed in the supine position. The patient's right upper extremity and bilateral groins were prepped and draped in standard fashion. Using ultrasound guidance, the right common femoral vein was accessed with a 21-gauge single-wall needle as part of a 7 French micropuncture kit. The needle was exchanged for a dilator, followed by a sheath. A baseline ACT was drawn then, and the sheath was connected to a continuous heparin saline flush.

Under fluoroscopic guidance, a 7 French BMX guide catheter was advanced over a 0.035" angled Glide guidewire through the inferior vena cava and superior vena cava into the distal right internal jugular vein. Next, under fluoroscopic guidance, a microcatheter was advanced over a microwire into the superior sagittal sinus. The microwire was then removed.

With the microcatheter in the superior sagittal sinus, PA and lateral projections demonstrated anterograde flow from the superior sagittal sinus into the right transverse sinus, sigmoid sinus, and internal jugular vein, with the right transverse sinus being dominant. Severe stenosis was noted involving the right distal transverse/sigmoid junction with stasis of contrast proximal to the stenosis.

The microcatheter was then attached to a manometer and calibrated. It was subsequently retracted under fluoroscopy to various positions, allowing time for pressure readings to equilibrate. The pressure readings in mmHg were as follows: superior sagittal sinus at 32 mmHg, right proximal transverse sinus at 31 mmHg, right distal transverse sinus at 31 mmHg, right proximal sigmoid sinus at 12 mmHg, and right distal sigmoid sinus at 12 mmHg.

The microcatheter was removed, and the guide catheter remained connected to a continuous heparinized saline flush. Weight-based heparin was administered intravenously at this time. Next, the guide catheter was advanced over the intermediate and microcatheter over the microwire through the right transverse and sigmoid sinuses into the superior sagittal sinus. The microwire was then removed.

With the microcatheter positioned in the superior sagittal sinus, PA and lateral projections showed anterograde flow into the right transverse sinus, sigmoid sinus, and internal jugular vein, alongside severe stenosis at the right transverse/sigmoid junction. Following this, a venous phase roadmap guided the advancement of the guide catheter over the intermediate and microcatheters into the proximal sigmoid sinus. After replacing the microwire with a balance heavyweight 14 exchange microwire, the microcatheter was removed while leaving the exchange microwire in place. Subsequently, an 8 x 80 mm stent was advanced over the microwire into the right transverse sinus and successfully deployed from the proximal right transverse sinus to the proximal sigmoid sinus, addressing the previously identified stenosis.

Following venous sinus stenting, post-stent angiography of the right common carotid artery in cervical view revealed unobstructed anterograde flow into the right common and internal carotid arteries, as well as the right external carotid artery with flow into its branches, including the superior thyroid, occipital, lingual, and facial arteries. There were no signs of atheromatous disease or significant stenosis. Similarly, post-stent angiography of the left common carotid artery in craniocervical view demonstrated anterograde flow into the left common and internal carotid arteries, extending into the intracranial segments of the left internal carotid artery and its branches such as the superior thyroid, occipital, lingual, facial, superficial temporal, and middle meningeal arteries. No aneurysms, arteriovenous malformations/fistulas, atheromatous disease, or significant stenosis were observed, affirming patent dural venous sinuses. Additionally, post-stent angiography of the left vertebral artery in cranial view confirmed anterograde flow into the left vertebral and basilar arteries, with collateral flow into the left posterior inferior cerebellar artery (PICA), bilateral anterior inferior cerebellar arteries (AICAs), superior cerebellar arteries (SCAs), and posterior cerebral arteries (PCAs). Retrograde flow in the right vertebral artery was noted, and no vascular abnormalities, significant stenosis, or vasculopathic changes were identified.

**Figure 7:**
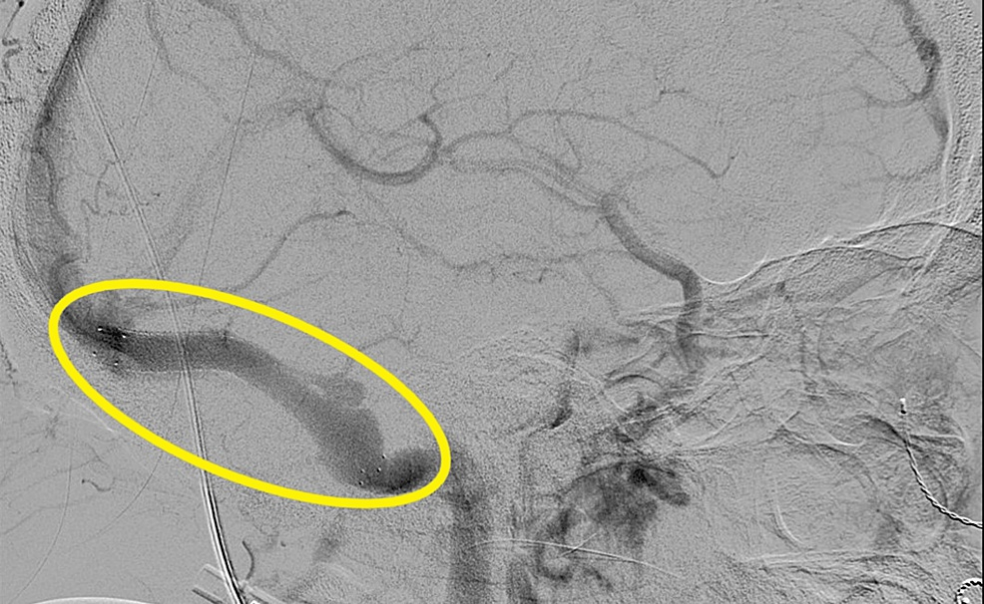
Perioperative fluoroscopy of right transverse-sigmoid sinus with an 8 x 80mm stent Yellow circle: 8 x 80mm stent located in right transverse-sigmoid sinus

A cerebral angiogram, venogram, venous manometry, and venous sinus stenting were performed by neurosurgery without any complications, revealing the following:

1) A selective cerebral angiogram and venogram demonstrated severe stenosis of the right transverse-sigmoid sinus junction with a significant venous gradient (Figure 6; yellow circle).

2) Successful selective catheterization and stenting of the right transverse-sigmoid sinus using an 8 x 80 mm stent, with significant improvement in the stenosis of the right transverse-sigmoid sinus on post-procedural angiography/venography (Figure 7; yellow circle).

Post-operatively, the patient was transferred to the surgical stepdown unit for closer monitoring with frequent neurological evaluations. Immediately following the procedure, the patient reported resolution of headache and neck pain, improvement in right-sided facial numbness, and unchanged blurry vision with a right lower quadrant visual field deficit. He was continued on dual antiplatelet therapy and started on dexamethasone. Acetazolamide dosing was increased to aid in visual symptoms.

The patient was discharged with the above medication regimen, including a planned dexamethasone taper over several days as well as outpatient ophthalmology follow-up for the ongoing visual field deficit. No changes in management were made by ophthalmology. At the 10-day postoperative follow-up appointment with neurosurgery, the patient reported some improvement in his visual field deficit with medication compliance. He did report mild intermittent right-sided frontal and occipital headaches but with overall improvement.

## Discussion

Chiari malformation type 1 is the most common among hindbrain abnormalities. Chiari malformation type 1 is defined as caudal, cerebellar, and/or brainstem herniation below the foramen magnum, specifically caudal tonsillar herniation below the level of the foramen magnum by at least 5 mm [8]. Our case demonstrated downward cerebellar tonsillar herniation extending up to 2.4 cm below the foramen magnum.

Dual antiplatelet therapy, comprising aspirin and clopidogrel, has been used as the primary strategy to reduce device-related thromboembolic complications. Based on the latest evidence, a combination of patient pharmacogenetics, drug response monitoring, and a patient-based approach to drug selection is both a predictive and cost-effective mechanism for achieving an individual’s target platelet inhibition levels [9]. Notably, since the start of venous sinus stenting, multiple changes have persisted in the anticoagulation protocol throughout various studies. All recent studies indicated appropriate dual antiplatelet therapy before stent placement, which includes testing P2Y12 and aspirin reaction unit (ARU) levels and discontinuing clopidogrel within the first six months of stent placement. Aspirin recommendations range from three months to lifetime use. Given the significant dissimilarity in the length of therapy and the lack of studies on modern P2Y12 inhibitors, further analysis is needed in the study of anticoagulation and venous sinus stenting [10].

The Chiari I malformation presents challenges in both its complications and management strategies. Complications associated with Chiari I malformation can include syringomyelia, hydrocephalus, and cranial nerve dysfunction, which often manifest as severe headaches, visual disturbances, and motor deficits. Management typically involves a multidisciplinary approach, including neurosurgery consultation for cases requiring surgical intervention. Surgical treatment options for C1M may include posterior fossa decompression to alleviate symptoms and prevent further progression of complications such as syrinx formation. The goal of management is to relieve symptoms related to cerebellar tonsillar herniation and improve cerebrospinal fluid dynamics, thereby reducing the risk of long-term neurological deficits. Complications involved in surgical decompression include pseudomeningocele, cerebrospinal fluid leakage from the wound site, infection, vascular injury, post-operative hydrocephalus, and medullary dysfunction [11]. Our case did not involve surgical decompression, but when dealing with venous sinus stenting, similar considerations must be applied regarding complications, including but not limited to infection, thrombosis, bleeding, and iatrogenic intracranial hypertension.

In a review of previous cases of posterior fossa decompression with or without duraplasty, ventral herniation was a commonly observed radiological finding. Within this subset population, and those with herniation greater than 5 mm, posterior fossa decompression with or without duraplasty was performed due to the progression of syrinx or worsening symptoms [12]. In another meta-analysis study evaluating the use of posterior fossa decompression with duraplasty, it was found that in the absence of hydrocephalus and cervical instability, posterior fossa decompression with duraplasty provided better outcomes but was associated with higher risk [13]. It is worth noting that, although the patient described suffered symptomatically in the setting of Chiari malformation type 1 and syringohydromyelia, the course was further complicated by severe stenosis of the right transverse-sigmoid sinus junction. In diagnosing idiopathic intracranial hypertension, transverse sinus stenosis appeared to yield the highest sensitivity and specificity in MRI-guided etiological association [14]. Overall, further studies need to be performed to evaluate an image-guided approach for intracranial hypertension, and the creation of clinical standardization in grading systems of Chiari malformation type 1 symptomatology needs to be established.

## Conclusions

In conclusion, understanding the complexities of Chiari malformation type 1 is crucial for effective management. Studies have shed light on its natural history, clinical presentations, and treatment options. Investigations into the pathophysiology of syringomyelia contribute to our understanding of Chiari malformation type 1 mechanisms. Updates on diagnosis and treatment strategies further refine therapeutic approaches. Imaging techniques play a significant role in accurate diagnosis, especially in cases of associated conditions like idiopathic intracranial hypertension. Continued research efforts, including meta-analyses on treatment outcomes, are vital for improving patient care and outcomes in Chiari malformation type 1.
